# A 2°C warming can double the frequency of extreme summer downpours in the Alps

**DOI:** 10.1038/s41612-025-01081-1

**Published:** 2025-06-19

**Authors:** Nadav Peleg, Marika Koukoula, Francesco Marra

**Affiliations:** 1 Institute of Earth Surface Dynamics, University of Lausanne, Lausanne, Switzerland; 2 Expertise Center for Climate Extremes, University of Lausanne, Lausanne, Switzerland; 3 Department of Geosciences, University of Padova, Padova, Italy

**Keywords:** Climate change, Climate-change impacts, Projection and prediction, Hydrology

## Abstract

Intense short-duration summer rainfall in mountainous areas can trigger a variety of natural hazards, including flash floods, debris flows, and urban floods. Warming is expected to intensify extreme sub-hourly rainfall events in response to an increased atmospheric water vapor content and invigorated storm dynamics. Here, we employ a new physically-based statistical model to estimate the projected intensification of sub-hourly and hourly extreme rainfall across 299 high-mountain Alpine stations in France, Germany, Switzerland, Italy, and Austria. Analyzing the projected intensification for 10-min rainfall at 1 to 3 °C of warming confirms a general intensification at a rate of 9% °C^−1^ over the Alpine region, with a stronger intensification at higher elevations. With a 2 °C increase in average regional temperature relative to the 1991–2020 period, extreme rainfall statistics over the Alps are likely to undergo significant changes, resulting in a two-fold increase in the probability of occurrence of the extreme rainfall levels used for infrastructure design and risk management.

## Introduction

Summer rainfall in the European Alps is often convective, associated with thunderstorms and substantial atmospheric instability^1^. This instability is exacerbated dynamically by the complex orography, which can produce very intense short-duration (minute-scale) rainfall bursts, adversely impacting the mountainous region and its surroundings. Natural hazards such as flash floods, debris flows, landslides, and urban floods may be triggered as a result of heavy rainfall events of this type, which can damage infrastructure and endanger lives^2^. One example is the extreme rainfall event that occurred in Lausanne (Switzerland) on the 11th of June 2018, with 41 mm of rainfall falling in just 10 min, an average rain rate exceeding 240 mm h^−1^ ^3^. In this extreme rainfall event, with an estimated return period of more than 300 years under present-day conditions, large parts of the city were flooded, resulting in damage costs of over 32 million euros. Another example is the extreme rainfall event that triggered multiple debris flows in the upper Adige river basin (Eastern Italian Alps) on the 16th of July 2009, during which 98 mm of rainfall were recorded in 1 h (exceeding the present-day 100-year return period)^4^, causing ~0.7 million euros in damage as well as altering the river’s morphology. In 2023, the estimated damage costs from natural hazards in Switzerland alone amounted to 77.5 million euros, with the largest share (66%) being attributed to landslides, followed by flooding (21%), rock avalanches (7%), and debris flows (6%), predominantly triggered by extreme rainfall (67%) and rain-on-snow events (18%)^5^.

Short-duration convective rainfall over Europe is foreseen to become more intense due to global warming^1,6,7^. This intensification is driven by an increased amount of water vapor dissolved in the air (~7% for every 1 °C increase in air temperature, according to the Clausius-Clapeyron (CC) relation), and by invigorated dynamics of the convective storms^8–10^. Consequently, although most of the Alps are projected to experience a decrease in summer rainfall amounts, storm frequency, and event duration^11^, extreme convective rainfall events (e.g., rainfall intensities exceeding the 99th percentile of present rainfall) are expected to become more frequent and intense in the future. Considering that the Alpine region is warming at a faster rate than other parts of the world^12,13^, the intensification of rainfall extremes could have serious consequences for triggering natural hazards, potentially affecting risk assessments, mitigation strategies, and climate change adaptation policies.

**Fig. 1:**
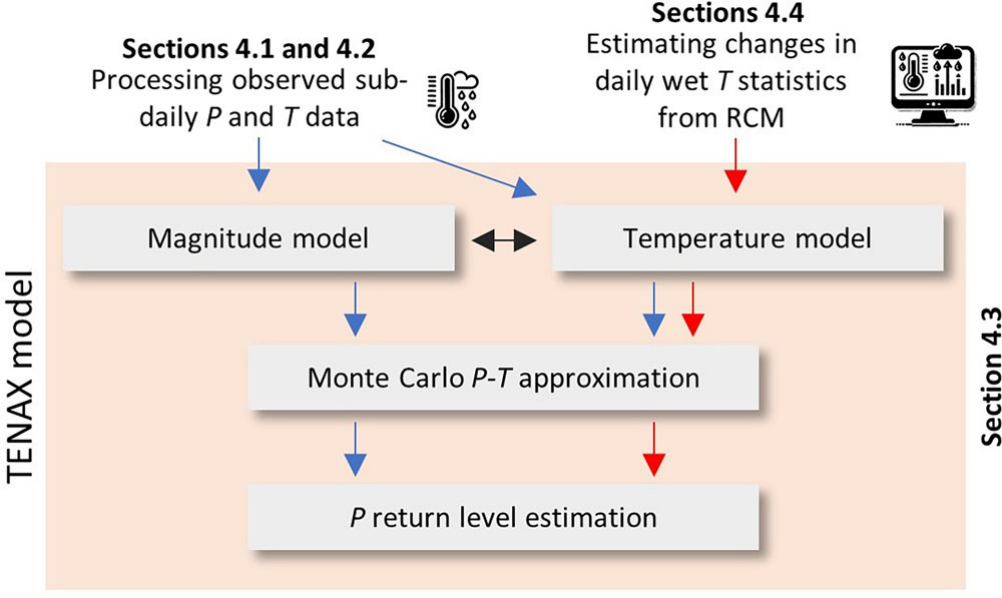
A schematic illustration of the TENAX framework to estimate changes in sub-daily rainfall return levels. *P* and *T* refer to precipitation and temperature.

Therefore, it is crucial to determine to what extent short-duration extreme rainfall will intensify as the climate warms^10^. Currently, the most advanced approaches to these evaluations are based on high-resolution convection-permitting models, capable of simulating rainfall at sub-daily and kilometer scales under varying warming scenarios^14,15^. Despite their importance in predicting changes in rainfall patterns, these models have difficulty predicting future short-duration extreme rainfall in complex mountainous regions^15,16^. Moreover, because of their extensive computational requirements, they often do not provide information at the sub-hourly resolution, are not available in all regions, and are not yet forced with the most updated global circulation models and emission scenarios. In a recent development, Marra et al.^17^ introduced TENAX, a physically-based model for the analysis of short-duration rainfall frequency, which has proven effective in predicting extreme sub-hourly rainfall intensification based on daily temperature shifts in the complex Alps environment. TENAX uses sub-daily rainfall and temperature data to quantify the statistical relationship between rainfall peaks and temperature and calculates rainfall return periods based on the observed distribution of temperatures during precipitation events. It consists of a magnitude model trained on observations only, which contains the physical dependence of heavy rainfall on temperature, and of a temperature model that describes the temperature during wet days. Regional climate models are then used to obtain projections of change in future temperature during wet days and their annual occurrence frequency. Assuming present-day rainfall-temperature relationships will persist in the future, it is possible to stochastically simulate rainfall peaks for future temperature conditions and project how sub-daily rainfall return levels will change (see Fig. 1 and “Methods”).

Here, we collected sub-hourly rainfall and air temperature records from 299 stations spanning the European Alps (including Austria, France, Germany, Italy, and Switzerland), and computed the change in frequency of summer short-duration rainfall extremes, recorded between June and September, under warming scenarios of 1 to 3 °C. The TENAX model was first used to condition rainfall intensities on the temperature at each station and then forced with the projected wet-day temperature changes from the EURO-CORDEX climate models. The results, presented next, indicate that extreme short-duration rainfall along the Alps will significantly intensify even if the regional temperature increases are limited to 1 °C. A 2 °C increase in the regional temperature may even double the frequency of today’s 50-year and 100-year storms.

## Results

### Extreme rainfall-temperature scaling

**Fig. 2:**
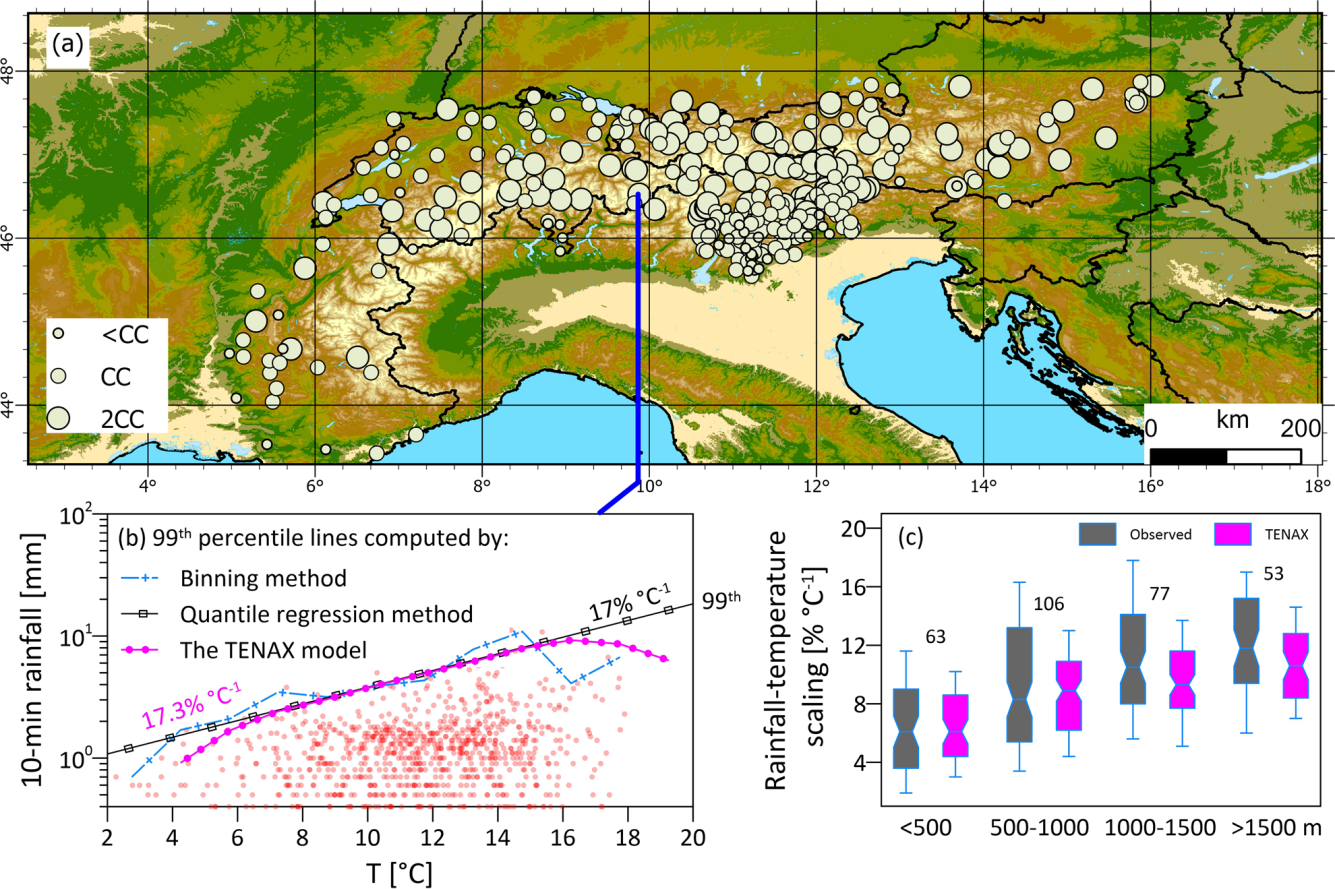
Overview of the climate stations and their associated rainfall–temperature relationships. **a** Observed extreme rainfall-temperature scaling rates for the 299 stations in the Alps at 10-min resolution. Symbol size differentiates between scaling rates lower (<CC), similar (CC), or approximately double (2CC) than the 7% °C^−1^ of the Clausius-Clapeyron scaling. **b** An example of the scaling rates estimated from the observed data (black line) and the TENAX model (purple line) for the Samedan station in Switzerland. **c** Observed (gray) and simulated (purple) scaling rates for different elevation bands. The center of the boxplot is the median, box edges are the quartiles, and whiskers represent the 10–90th percentiles. Numbers represent the sample size.

We first plot the observed summer scaling rates between the 99th percentile 10-min rainfall extremes and the temperature over the 24 h preceding the peak of the rainfall event for the 299 stations in the Alps (Fig. 2a). We confirm that most of the stations (146) follow the expected theoretical 7% °C^−1^ CC intensification^8^, while other stations (117) are characterized with double-CC rates [i.e., around 14% °C^−1^,^9^], and a few (36) with scaling rates smaller than 7% °C^−1^. The distribution of scaling rates over the Alps exhibits a spatial pattern that is less apparent when plotted on a map (Fig. 2a) but becomes more evident when the stations are clustered by elevation (Fig. 2c). The scaling rates were found to increase with elevation, from 6.2% °C^−1^ on average for stations located below 500 m to 12% °C^−1^ on average for stations situated above 1500 m (Fig. 2c). Consequently, high mountainous areas are expected to experience greater rainfall intensification rates than lower-elevation regions. This is intriguing as it opposes the “reverse orographic effect” observed in the Alps^18,19^, where extreme short-duration rainfall intensities tend to decrease with increasing elevation, possibly indicating a weakening of this effect in the future.

We then compared the observed and TENAX-simulated scaling rates. As an example, a comparison of scaling rates for a central Alpine station is presented in Fig. 2b (scaling rates of all stations are provided in the Supplementary Table S1). When comparing all stations, a small deviation of 0.8% °C^−1^ (R^2^ of 0.5) is found, implying that the simulated values are slightly lower than the observed values, likely due to the censoring of weak events operated by the model (see “Estimating rainfall return levels” and^20^). The deviation varies with elevation; TENAX marginally overestimates scaling rates at elevations less than 500 m by 0.3% °C^−1^, whereas at elevations above 1500 m, the model underestimates scaling rates on average by 1.32% °C^−1^ (Fig. 2c)—yet still pointing on larger scaling rates at higher elevations.

We assume that the present-day rainfall-temperature relationship remains stationary and will persist in the future (Methods and^4^). While our findings indicate scaling rates close to theoretical values, we acknowledge that this is not observed everywhere^21,22^, and some studies suggest that the current scaling may not hold under future climate conditions^21,23,24^. However, in the Alpine region, this assumption is well-supported. Several studies have demonstrated that near CC scaling of 7% °C^−1^ is observed both in present-day data^20,22^ and in future projections using regional convection-permitting models^25–27^. Recent studies have shown that, both in recent decades and under future climate conditions, the response of rainfall convection to warming is the dominant driver of summer rainfall intensification, exceeding the influence of atmospheric dynamics in the region^28,29^. Yet, atmospheric dynamics remain an important consideration as they are expected to change in the region^16^, and are also statistically represented within our modeling framework (see “Estimating rainfall return levels”).

### Predicting changes in rainfall extremes

**Fig. 3:**
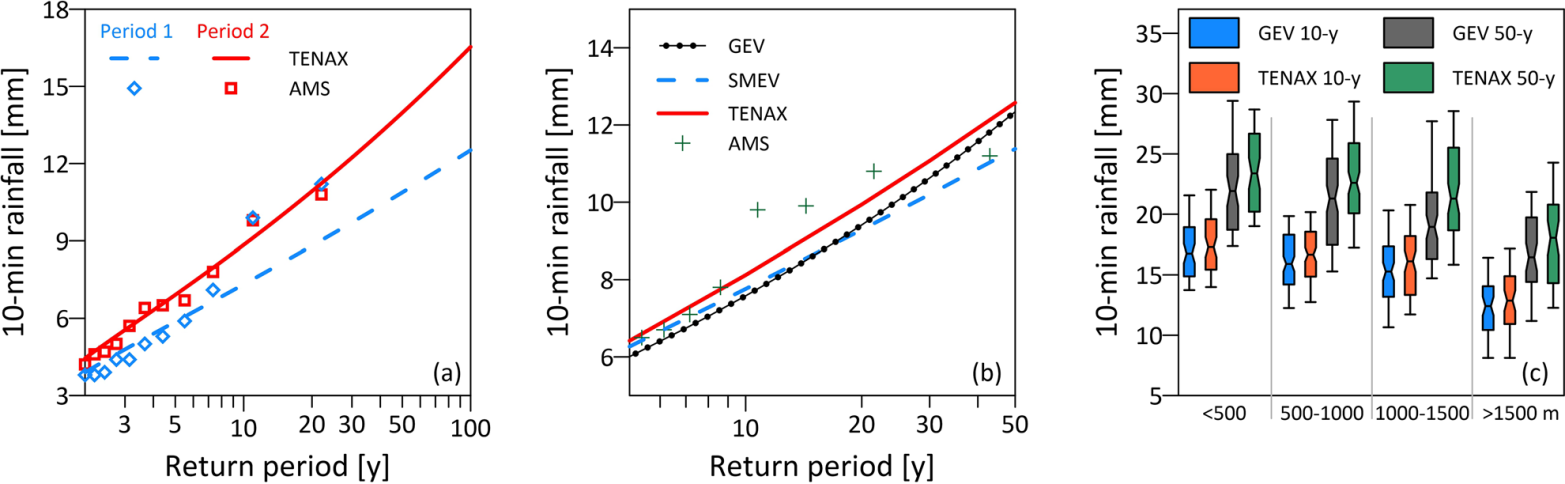
TENAX evaluation and projection of short-duration rainfall extremes. **a** Empirical 10-min annual maxima (blue diamond symbols) and the computed return levels by TENAX (blue dashed line) fitted for the Samedan station in Switzerland for the period 1981–2001. Additionally, the annual maxima (red square symbols) and the computed return levels by TENAX (red solid line) are projected based on temperature shift only to the period 2002–2022. **b** Comparison between the 10-min annual maxima (plus symbols), GEV-based (black dotted line), SMEV-based (dashed blue line), and TENAX-based (red solid line) return levels for the Samedan station for the period 1981–2022. **c** Comparison of 10- and 50-year GEV-based (blue and gray boxplots) and TENAX-based (orange and green boxplots) return levels for all stations and for different elevation bands.

To exemplify TENAX’s abilities in predicting changes in hourly and sub-hourly summer extreme rainfall intensities with temperature shifts, we focused on the same climate station presented in Fig. 2b (Samedan). We divided its 42-year record into two equal periods and applied the TENAX model to the first period from 1981 to 2001, estimating rainfall return levels up to a 100-year return period and comparing them qualitatively with the empirical annual maxima (blue dashed line and diamond symbols in Fig. 3a). Then, estimating the temperature shift between the two periods (see “Climate projections”), and without modifying the TENAX parameters, we estimated the return levels for the period 2002–2022 (red square symbols and line in Fig. 3a), finding a close match between the estimates. These return levels are obtained without using any information on sub-daily rainfall from the second period (Fig. S1 presents a similar analysis for additional stations). To further assess the ability of the model to predict rainfall return levels, we compared our projections with GEV- and SMEV-based methods^30^ that were computed for the complete 42-year record (Fig. 3b). Overall, all three methods have a high degree of overlap, with TENAX projections being well aligned with empirical annual maxima for this station (Fig. 3b and^17^).

To quantify the performance of TENAX at estimating rainfall return levels in comparison to a traditional method, we computed the return levels for all stations using both GEV and TENAX and plotted them as a function of elevation (Fig. 3c). Extreme rainfall intensities diminish with elevation (especially at stations above 1500 m) following a reverse orographic effect^18^, which is captured by both TENAX and GEV methods at 10-min and hourly scales. When comparing the return levels for all stations for 10-min duration, a consistent but rather small overestimation by TENAX compared to GEV is found (Fig. 3c); 0.57 mm (R^2^ of 0.88) for the 10-year and 1.5 mm (R^2^ of 0.47) for the and 50-year return periods. For the 10- and 50-year return periods, the bias is largest between 500 and 1500 m (0.66 and 1.75 mm, respectively), and drops sharply at the low (0.35 and 0.53 mm) and high (0.53 and 0.98 mm) elevations. Keeping in mind that the GEV estimates are subject to relatively larger stochastic uncertainties than TENAX^17^, the reduced agreement at 50-year return periods can be partially due to noise in the GEV estimates. In fact, GEV estimates are not the ground truth and are here used to demonstrate plausible ranges and dynamics of the return levels in the Alps. It can be concluded that the TENAX estimates are at least equally realistic and physically sound.

### Future return periods

**Fig. 4:**
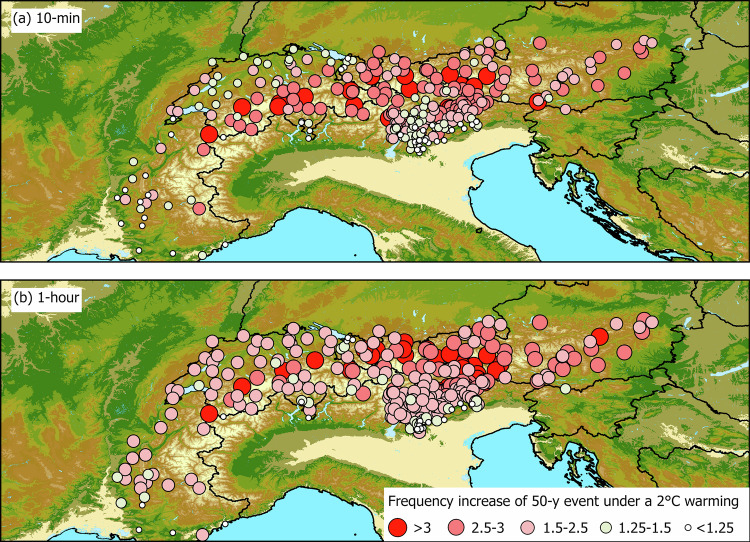
Projected increase in the frequency of extremes equivalent to today’s 50-year return levels under a 2 °C warming scenario. **a** 10 min duration and **b** 1 h duration.

For each station, we computed the 5- to 100-year future rainfall return levels for warming scenarios of 1 °C, 2 °C, and 3 °C over the Alps, based on regional climate model projections (see “Climate projections”), for the 10-min and hourly durations. The results for individual stations are summarized in the Supplementary Table S2, and here we present the key findings for the intensification of short-duration summer rainfall across the region. Focusing on the changes to the 50-year rainfall return levels, that is, levels high enough to be relevant for the design of infrastructure and risk management, for the 2 °C warming scenario as an example, we can identify a considerable increase in the frequency of future return periods at 10-min (Fig. 4a) and hourly (Fig. 4b) durations. In some cases, today’s 50-year events are projected to triple their frequency, meaning that the same extreme rainfall intensity that occurs today on average every 50 years will occur on average every 16 years in the future. As expected from the spatial distribution of the scaling rates (Fig. 2a), extreme short-duration rainfall intensifies more at high elevations, resulting in higher factors of change in the frequencies of return periods (Figs. 4 and 6). The projected intensification of 10-min extremes is consistently greater than the one of hourly extremes (Fig. 4), implying that the information we can obtain from high-resolution climate models is still insufficient to properly quantify the future risks related to extreme downpours.

**Fig. 5:**
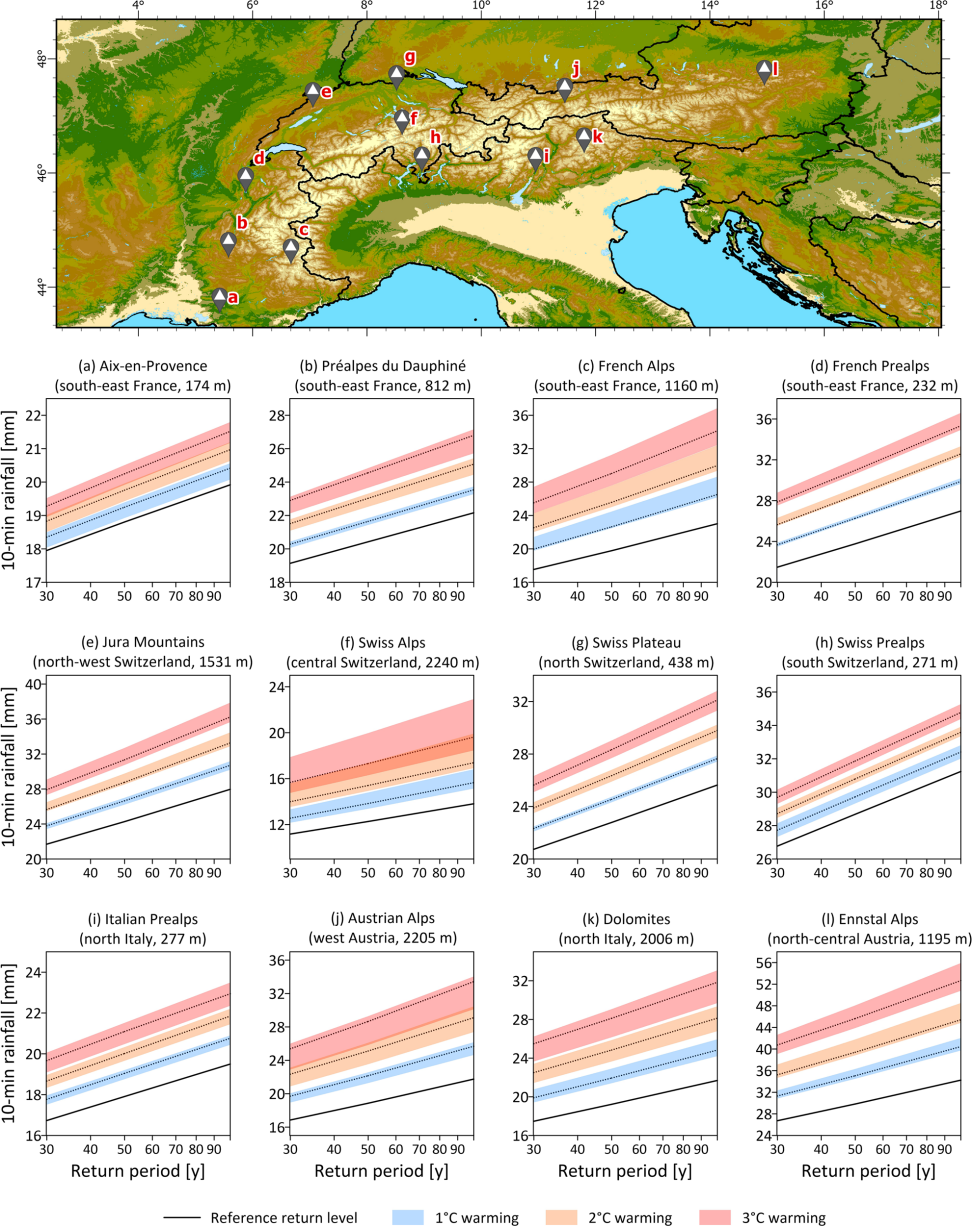
Present (1991–2020; black solid lines) and projected future return levels for warming scenarios of 1 °C (blue), 2 °C (orange), and 3 °C (red) at 12 selected stations along the Alps. **a** Aix-en-Provence, **b** Prealpes du Dauphine, **c** French Alps, **d** French Prealps, **e** Jura Mountains, **f** Swiss Alps, **g** Swiss Plateau, **h** Swiss Prealps, **i** Italian Prealps, **j** Austrian Alps, **k** Dolomites, and **l** Enstal Alps. Shaded areas indicate the 10th–90th percentile range derived from the climate model ensemble.

**Fig. 6:**
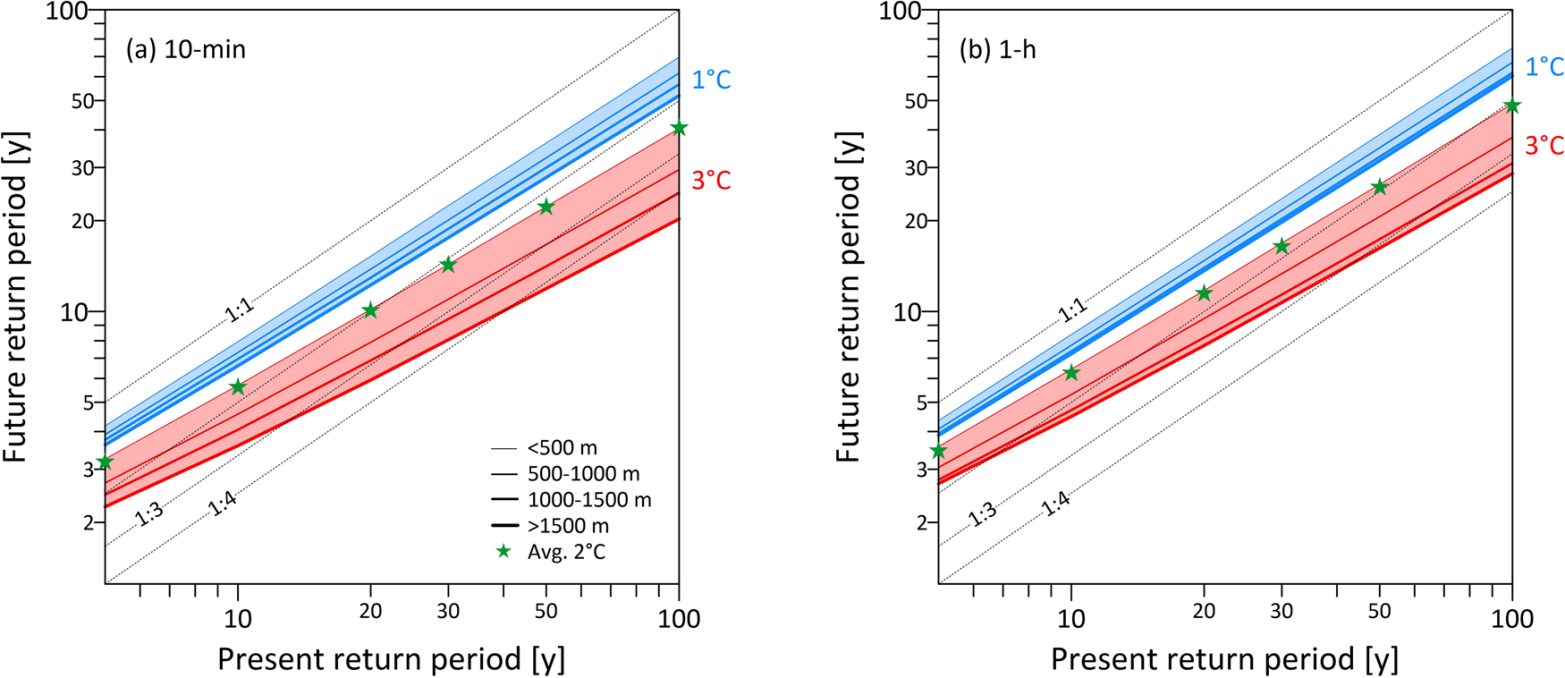
Assessments of short-duration rainfall intensification in the Alps. Projected future return periods compared to current return periods under 1 °C (blue) and 3 °C (red) warming scenarios for (**a**) 10 min and (**b**) 1 h durations. Line thickness represents the elevation bands, from below 500 m (thin lines) to over 1500 m (thick lines). Changes in return periods over the Alps for 2 °C (red) warming scenarios are presented using star symbols, which represent averages across all elevations and stations in the region.

While the results presented in Fig. 4 (and later in Fig. 6) focus on changes in rainfall return levels based on the ensemble multi-model median, we recognize the substantial uncertainties associated with climate projections and systematically assess and quantify them (“Data and model uncertainty”). Even when considering the uncertainties emerging from the climate models (Fig. 5 and S5), the trends of change in rainfall return periods along the Alps remain evident. It can be observed that, although the absolute range of uncertainties varies across stations and regions, they consistently increase as a function of both the warming scenarios and the return period. Additionally, uncertainty increases with elevation (Fig. 5 and S5b), with rainfall return level forecasts for stations above 1500 m exhibiting significantly greater uncertainty than those at lower elevations. This aligns with expectations, as complex topographies tend to introduce larger variations in projections from regional climate models. Nevertheless, despite the substantial uncertainties detected, particularly along the Alpine main ridge (Fig. 5c, f, j, k), future rainfall intensities are projected to increase significantly beyond current rainfall return levels.

To further investigate the level of intensification, we plotted the future return periods for different warming scenarios (1 °C and 3 °C) compared to the present return periods for the 10-min (Fig. 6a) and 1-h (Fig. 6b) durations averaged across four elevation bands. We found that the variability of the change in the future return periods with elevation increases with warming level and rainfall intensity, but decreases with duration; this means that the largest differences in short-duration rainfall intensification between low and high elevations are found for the 100-year present return level for the 3 °C warming scenario and 10-min duration. Extreme rainfall at 10-min duration is generally associated with a greater degree of intensification than at hourly duration. For example, for the 3 °C warming, some future return periods at the 10-min duration cross the 1:4 factor line, meaning that their frequency is projected to increase by four-fold; the same is not observed at hourly duration. The green star symbols in Fig. 6 represent the average changes in future return periods along the Alps considering all stations at all altitudes under the 2 °C warming scenario. For both 10-min (Fig. 6a) and hourly (Fig. 6b) durations, the 2 °C changes in return periods closely follow the 1:2 factor line, implying a doubling of the frequency of occurrence of today’s extreme rainfall intensities under 2 °C warming.

## Discussion

Today’s infrastructure is typically designed to withstand extreme short-duration rainfall events based on return periods that vary by region, infrastructure type, and local risk assessments. Urban drainage systems, for example, are typically designed to prevent floods for 10- to 30-year rainfall return periods, depending on local regulations and area characteristics (e.g., residential versus commercial zones)^31^. Larger cities or critical areas might use higher standards, such as 50-year return periods^32^. It is common for transportation infrastructure (e.g., roads, bridges) to be designed to endure rainfall return periods of 50 to 100 years, and critical infrastructure like major highways, railways, and airports might be designed to tolerate even more severe and rare rainfall events^33^.

The intensification of weather extremes caused by global warming will likely significantly increase the frequency and severity of natural hazards affecting contemporary infrastructure^34,35^. The economic and social implications of more frequent natural hazards are far-reaching; natural disaster damage costs are already increasing steadily over time^36^ and will likely continue to rise as extreme rainfall intensifies, placing a growing burden on communities and economies. Given the anticipated doubling or even tripling of extreme short-duration rainfall frequency in some Alpine areas (Fig. 6), we call for an immediate reevaluation of regional infrastructure resilience standards. Our findings emphasize the need to promptly update engineering and urban planning standards that still rely on historical climate data. Adapting to the projected increase in extreme event frequency, particularly in high-elevation regions, is essential to prepare urban drainage systems and other critical infrastructure for intensified rainfall. Proactive revisions to resilience guidelines and risk management procedures can help mitigate potential risks, safeguarding communities and economies.

Our findings highlight the importance of limiting global warming to 1.5 °C or lower from current levels (i.e., relative to the 1991–2020 reference period). A 2 °C warming scenario may lead to a substantial intensification of short-duration extreme rainfall, potentially doubling the frequencies of life-threatening events caused by extreme rainfall (Fig. 6). Current commitments^37^, including those made in Glasgow, suggest emission pathways that could stabilize the rise in global temperature between 1.6 °C and 1.8 °C above pre-industrial levels^38^ (i.e., relative to 1850–1900). Since global temperatures have already increased by 1.2 °C from pre-industrial levels to the reference period^39^, and given that the region is warming at a faster rate than the global average^13^, this suggests a potential further increase close to 1 °C if current commitments are met. This underscores the need for sustained and effective efforts to reduce greenhouse gas emissions and meet current targets, as mitigating can help limit the most severe impacts on the frequency and intensity of extreme rainfall, thereby reducing pressure on infrastructure, ecosystems, and human systems.

## Methods

### Extreme rainfall and temperature sampling

We analyzed summer rainfall (that is, rainfall recorded between June and September) for 299 climate stations located along the Alps (Table S3). To examine the extreme rainfall, we applied the unified framework proposed by ref. ^40^ to identify and sample independent rainfall peaks from the data, following a two-step process. First, independent “rainfall events” are defined as rainfall periods separated by at least 24-h dry intervals, ensuring that the events are temporally isolated and statistically independent. Second, rainfall peaks of duration *d* are defined as the duration maxima of each rainfall event using a running window approach. The window size *d* corresponds to the duration of interest, and the time step is set to match the temporal resolution of the rainfall data. This method guarantees that the selected rainfall peaks share the statistical properties of the annual maxima for the same duration, as demonstrated in previous studies (e.g.,^17,40,41^). This approach provides a robust framework for consistent event identification across varying durations, making it suitable for the analysis of extreme rainfall events across durations—in our case, *d* was set to hourly and 10-min scales.

Rainfall records have time intervals of 5, 6, and 10 min (Table S3) and were homogenized to 10- or 12-min (for the 5- and 6-min stations, respectively) through aggregation. For each station, years with more than 10% missing records were excluded from the analysis. Additionally, as described by ref. ^17^, we sampled and averaged the temperature for the 24 h preceding each peak to identify the associated proxy temperature.

### Computing empirical scaling rates

The scaling rates between extreme rainfall and temperature are computed using a quantile regression model^42^ that is fitted to the logarithm of the *q* percentile of precipitation *P*_*q*_ as a function of near-surface air temperature *T*:

1 $$\log ({P}_{q})=\alpha +\beta T,$$

from which the scaling rate is derived:

2 $$\frac{\partial {P}_{q}}{\partial T}=({e}^{\beta }-1)\cdot 100.$$

### Estimating rainfall return levels

We estimate rainfall return levels using the TEmperature-dependent Non-Asymptotic statistical model for eXtreme return levels (TENAX)^17^, which enables assessing changes in short-duration precipitation extremes as a function of shifts in near-surface air temperature during wet days. TENAX separates the dependence of extreme precipitation on temperature from the occurrence of precipitation events and combines this information to estimate precipitation return levels. TENAX is composed of three components: (i) a magnitude component that models the exceedance probability of rainfall peaks as a function of temperature; (ii) a temperature component that models the distribution of temperature associated with the rainfall peaks; (iii) a return level estimation component that estimates return levels based on the first two components. The magnitude *x* of extreme rainfall peaks for a given duration is modeled using a Weibull distribution, with parameters dependent on near-surface air temperature *T*:

3 $$W(x;T)=1-\exp \left[-{\left(\frac{x}{{\lambda }_{0}\cdot {e}^{aT}}\right)}^{{\kappa }_{0}+bT}\right],$$

where *λ*_0_ and *a* describe the exponential dependence of the scale parameter on temperature (thus conceptualizing based on scaling rates), and *κ*_0_ and *b* describe the temperature-dependent shape parameter. A likelihood ratio test run over all the climate stations showed that in most cases *b* is not significantly different from zero.

The distribution of temperatures during precipitation events is here modeled using a normal distribution with parameters *μ* and *σ*, which describe the mean and standard deviation of near-surface air temperature of the 24 h preceding the peak of the events^17^. Once the magnitude *W*(*x*; *T*) and the temperature *g*(*T*) components are established, a stochastic approach is used to generate a large collection of temperatures *T*_*i*_ with *i* = 1, …, *N* sampled from *g*(*T*), to obtain a Monte Carlo approximation of the marginal cumulative distribution function $F(x)={\int}_{{\mathcal{T}}}W(x;T)g(T)\,dT$, where ${\mathcal{T}}$ is the domain of *g*(*T*). An estimate of the distribution of annual maxima *G*(*x*) can then be obtained as^30^:

4 $${G}_{{\rm{TENAX}}}(x)\simeq {\left(\frac{1}{N}\mathop{\sum }\limits_{i = 1}^{N}W(x;{T}_{i})\right)}^{n},$$

where *N* is the number of stochastically generated events, and *n* is the average number of independent events per year. Precipitation return levels are then computed by inverting the above equation^17^ and^41^ provide additional information on the TENAX model, its parameterization, calibration, validation procedure, uncertainties, and examples of its application.

### Climate projections

The main assumption behind the estimation of future rainfall return levels using TENAX is that the magnitude component described above, which represents the statistics of convective rainfall in the location of interest at a given temperature, is invariant. This means that we assume the influence of other covariates (e.g., aerosol concentration) on precipitation intensity at a given temperature to be negligible. We tested this assumption in hindcast by splitting the record of each climate station with records longer than 30 years (74 stations) into two periods of equal length and estimated *W*(*x*; *T*) separately for the two periods, as presented in Fig. 3a for the Samedan station and in Fig. S1 for nine other stations along the Alps. We then tested whether the null hypothesis of having a unique model estimated for the entire period, as opposed to the alternative hypothesis of having two models, could be rejected using a likelihood ratio test at a 5% significance level. We found that the null hypothesis could be rejected in only 6 out of 299 stations.

To parameterize TENAX for future rainfall return periods, it is necessary to collect information on the changes in the mean Δ*μ* and standard deviation *δσ* of temperature during wet days and the change in the total number of annual rainfall events *δn*^17^. This information was obtained from the “historical” and “RCP8.5” simulations of 17 regional climate models (summarized in Table S4) covering the Alps. While the use of CMIP5-EURO-CORDEX (GCM, downscaled by a RCM) to assess changes in temperature and precipitation has been well established^43^, the models exhibit known uncertainties stemming from their physical formulations, the omission of certain atmospheric processes, and the inherited uncertainties from global circulation models^43,44^. Notably, these models tend to simulate too cold and too wet climates due to factors such as excessive snow accumulation and albedo effects, overestimated cloud cover, underestimated evapotranspiration, and deficiencies in convective parameterization^45^. For example, summer temperatures can be underestimated by ~0.5 °C due to misrepresented aerosol effects^46^. In the Alps, the models simulate colder summer temperatures (by 0.8 °C) and a higher frequency of rainfall events (by 14.8%) compared to observations^47^. We account for these climate model uncertainties in our predictions of changes in extreme short-duration rainfall, as discussed in “Data and model uncertainty”.

We depart from the conventional use of emission scenarios and assess how a fixed increase in near-surface air temperature over the EURO-CORDEX domain affects Δ*μ*, *δσ*, and *δn*. This approach isolates temperature change as the primary driver, independent of specific emission pathways (i.e., solely as a function of general warming levels, see ref. ^48^ for further discussion). Our first step was to calculate *μ*, *σ*, and *n* for each climate model for the historical period 1976–2005 for all grid cells intersecting with one of the climate stations in the Alpine domain. In addition, we computed the historical mean daily near-surface air temperature *T*_*a*_ for each grid. Then, using a moving average window for the projected period 2006–2100, we calculated decadal values of future *μ*^*F*^, *σ*^*F*^, *n*^*F*^, and ${T}_{a}^{F}$. A power equation was then used to link the change in temperature during wet days Δ*μ* and the shift in near-surface air temperature Δ*T*_*a*_:

5 $$\Delta \mu ={c}_{1}\cdot \Delta {T}_{a}^{{c}_{2}}+{c}_{3},$$

where Δ*μ* = *μ*^*F*^ − *μ*, $\Delta {T}_{a}={T}_{a}^{F}-{T}_{a}$, and *c*_1...3_ are the regression coefficients. This enabled finding the required value of Δ*μ* for the specific cases of increase in 1 °C, 2 °C, and 3 °C of near-surface air temperature.

The regressions of *δσ* and *δn* with Δ*Ta* were also fitted using power relations:

6 $$\delta \sigma ={c}_{1}\cdot \Delta {T}_{a}^{{c}_{2}}+1,$$

and

7 $$\delta n={c}_{1}\cdot \Delta {T}_{a}^{{c}_{2}}+1,$$

where $\delta \sigma =\frac{{\sigma }^{F}}{\sigma }$ and $\delta n=\frac{{n}^{F}}{n}$, and *c*_1_ and *c*_2_ being the coefficients. We computed the R^2^ goodness-of-fit for each grid cell for these three regressions and preserved only those fits exceeding 0.75 (52% of all fits). The median changes in Δ*μ*, Δ*σ*, and *δ*n for the 1–3 °C increase in regional temperature are presented in Fig. S2–S4.

### Data and model uncertainty

The climate station data we used varies in both temporal scales and recording periods, contributing to uneven uncertainty levels in rainfall return level estimates across stations. While some stations provide extensive records spanning up to 42 years (from Switzerland; over the central Alps), others cover only 15 years (Austria); this is still within a reasonable length of data to apply the TENAX model to estimate return levels, but wider uncertainties are to be expected in some regions^17,30^. Moreover, the stations recorded data over different time intervals and we treat the longest period covering 1981–2023 as the “present” period. While the TENAX model does not need stationarity assumptions for its training, we assume the climate to be stationary over this period in computing the changes in wet-day air temperature and rainfall occurrence derived from climate models to generate the future rainfall return periods.

The application of the TENAX model for estimating future rainfall return periods involves several layers of uncertainty. First, uncertainties may accumulate throughout the stepwise process of translating climate model outputs into temperature and rainfall frequency change factors, considering both the transition from daily to sub-daily scales (see ref. ^17^ for a detailed discussion on the topic) and the relatively coarse resolution of the climate models (11 km), which may not adequately represent local climate conditions (particularly in complex terrains). In addition, the model’s reliance on time-invariant temperature-precipitation scaling, while widely supported^10^ and tested at the station level in hindcast, may not hold exactly in the future considering potential changes in climate dynamics^49^ (though this seems unlikely^26^) and that future wet-day temperatures may reach values unexplored in the observations.

Given the potential large uncertainties in climate projections, we have quantified them alongside presenting the change in the multi-model median. First, we calculated the change in rainfall return levels for the individual climate projections, as illustrated for the 17 climate models and three warming scenarios at the Samedan station (Fig. S5a). Next, we calculated the 10–90th percentile range for each return period, defining it as the plausible uncertainty range derived from the ensemble of climate trajectories. We applied this procedure to all stations (see selected 12 in Fig. 5) and found that uncertainties increase with both warming and return period (see Results for details). Further, we summarized the 10–90th percentiles of the ratio between the standard deviation and mean signal change from the ensemble of climate projection per station to illustrate the uncertainties arising from climate model uncertainty in future rainfall return levels as a function of warming scenarios, return periods, and elevation (Fig. S5b).

## Supplementary information

Supplementary information

Supplementary table 1

Supplementary table 2

Supplementary table 3

Supplementary table 4

## Data Availability

Precipitation and temperature data are available from a variety of sources: Switzerland - MeteoSwiss (https://www.meteoswiss.admin.ch/), Austria - GeoSphere Austria (https://data.hub.geosphere.at/dataset/klima-v1-10min), France - Météo-France (meteo.data.gouv.fr), and Italy - Agenzia Regionale per la Prevenzione e Protezione Ambientale del Veneto (https://www.arpa.veneto.it/), Provincia Autonoma di Trento (https://www.provincia.tn.it/), and Provincia Autonoma di Bolzano (https://meteo.provincia.bz.it/default.asp). Data of the regional climate models (summarized in Table S4) are available on the EURO-CORDEX website at https://www.euro-cordex.net/.

## Code availability

The TENAX model is available at 10.5281/zenodo.8332232.
