# Supplementary material for: A 2°C warming can double the frequency of extreme summer downpours in the Alps
Source: NPJ Clim Atmos Sci. 2025 Jun 19;8(1):216. doi: 10.1038/s41612-025-01081-1 (PMC12178906; doi:10.1038/s41612-025-01081-1)
Supplement: Supplementary file 1 — Supplementary information [file 41612_2025_1081_MOESM1_ESM.pdf]

A 2°C warming can double the frequency of extreme sub-hourly rainfall in the Alps

Nadav Peleg[1,2*†], Marika Koukoula[1†] and Francesco Marra[3†]

[1*]Institute of Earth Surface Dynamics, University of Lausanne, UNIL-Mouline, Lausanne, 1015, Switzerland.
[2]Expertise Center for Climate Extremes, University of Lausanne, UNIL-Mouline, Lausanne, 1015, Switzerland.
[3]Department of Geosciences, University of Padova, Via Gradenigo 6, Padova, 35131, Italy.

*Corresponding author(s). E-mail(s): nadav.peleg@unil.ch;
Contributing authors: marika.koukoula@unil.ch;
francesco.marra@unipd.it;
[†]These authors contributed equally to this work.

Supplementary material

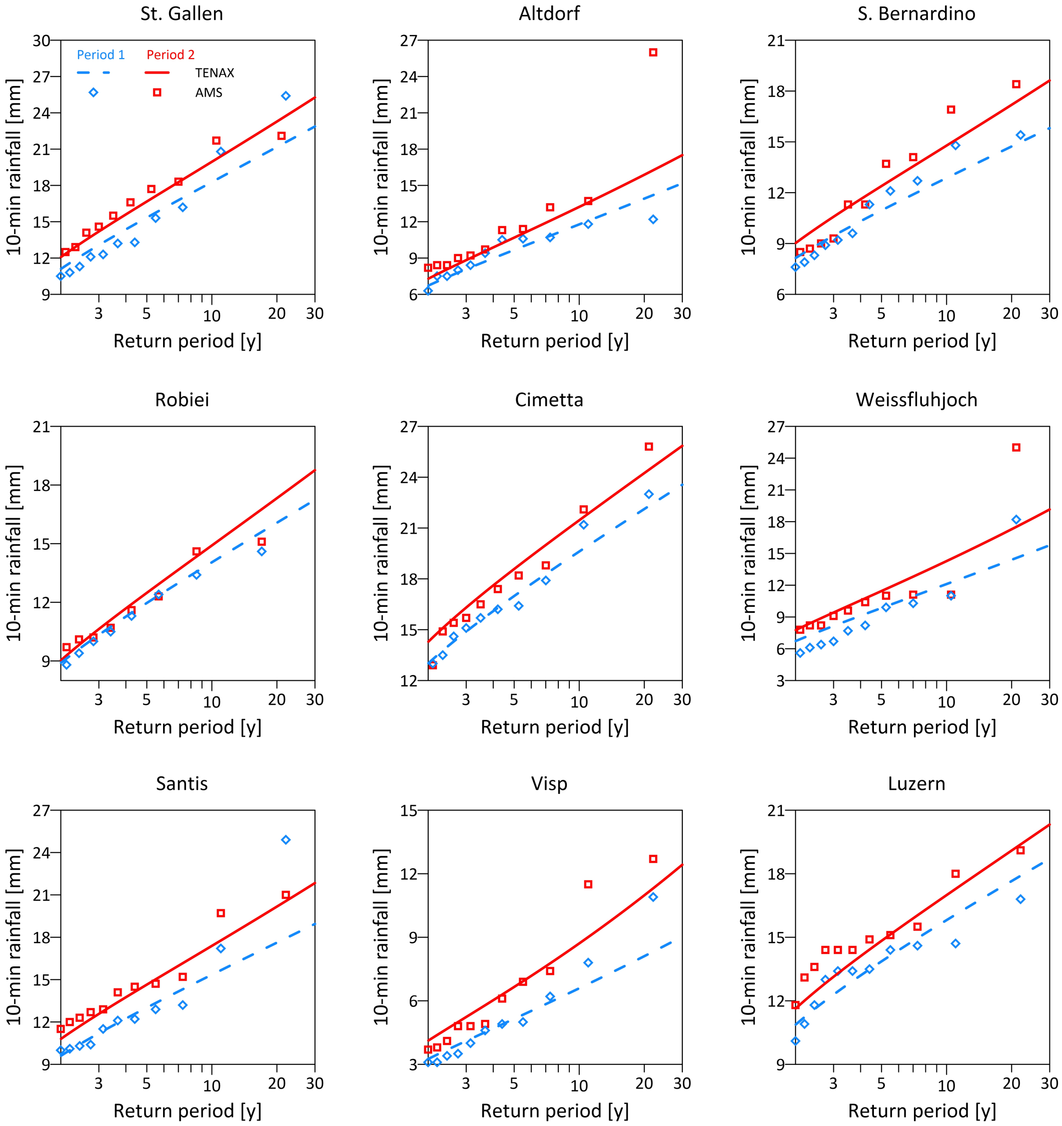

Fig. S1 Empirical 10-min annual maxima (blue diamond symbols) and the computed return levels by TENAX (blue dashed line) fitted for the nine stations in Switzerland for the period 1981-2001. The annual maxima (red square symbols) and the computed return levels by TENAX (red solid line) are projected based on temperature shifts only to 2002-2022.

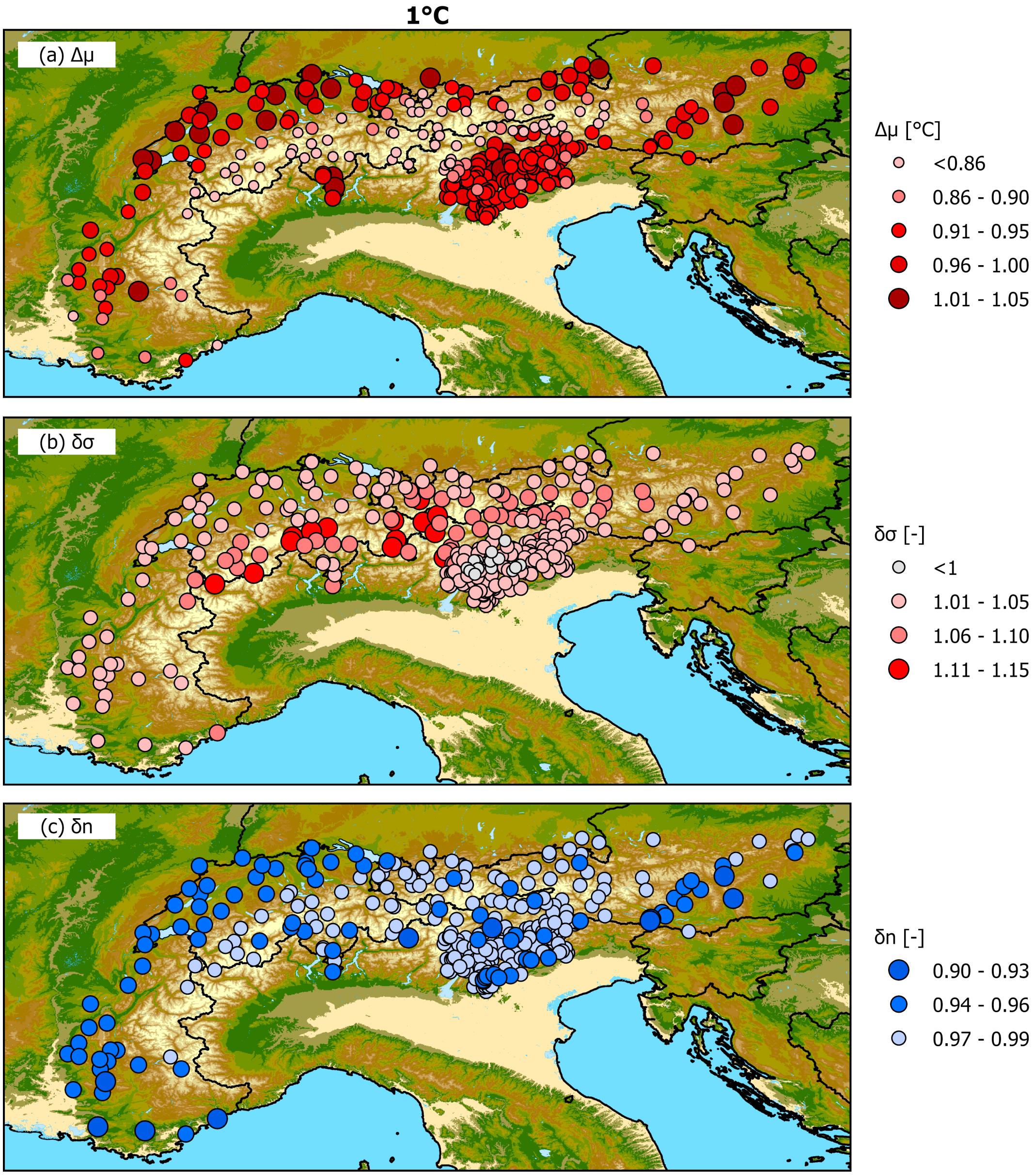

Fig. S2 Median changes in $\Delta\mu$, $\Delta\sigma$, and δn for the 1°C increase in regional temperature.

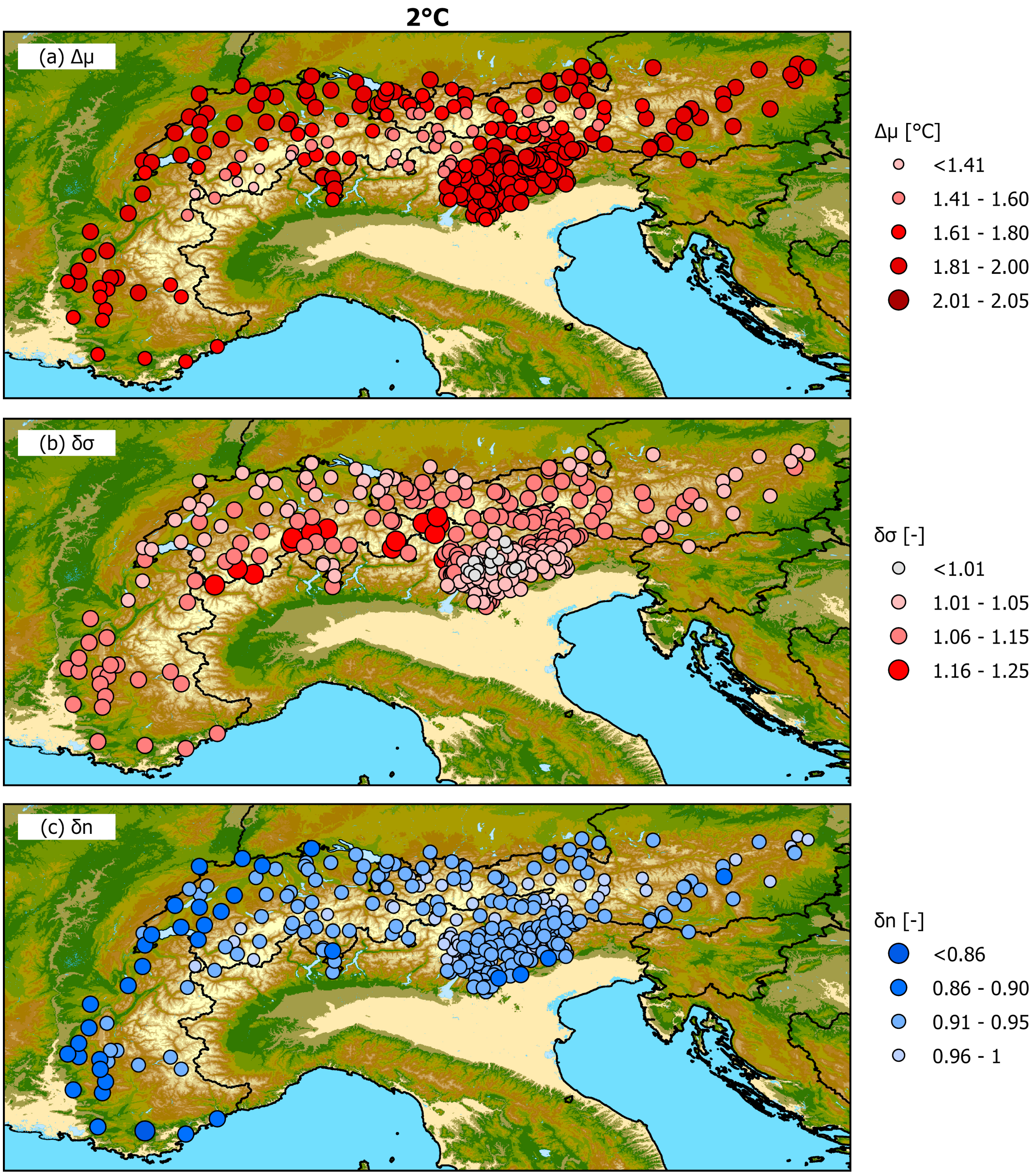

Fig. S3 Median changes in $\Delta\mu$, $\Delta\sigma$, and δn for the 2°C increase in regional temperature.

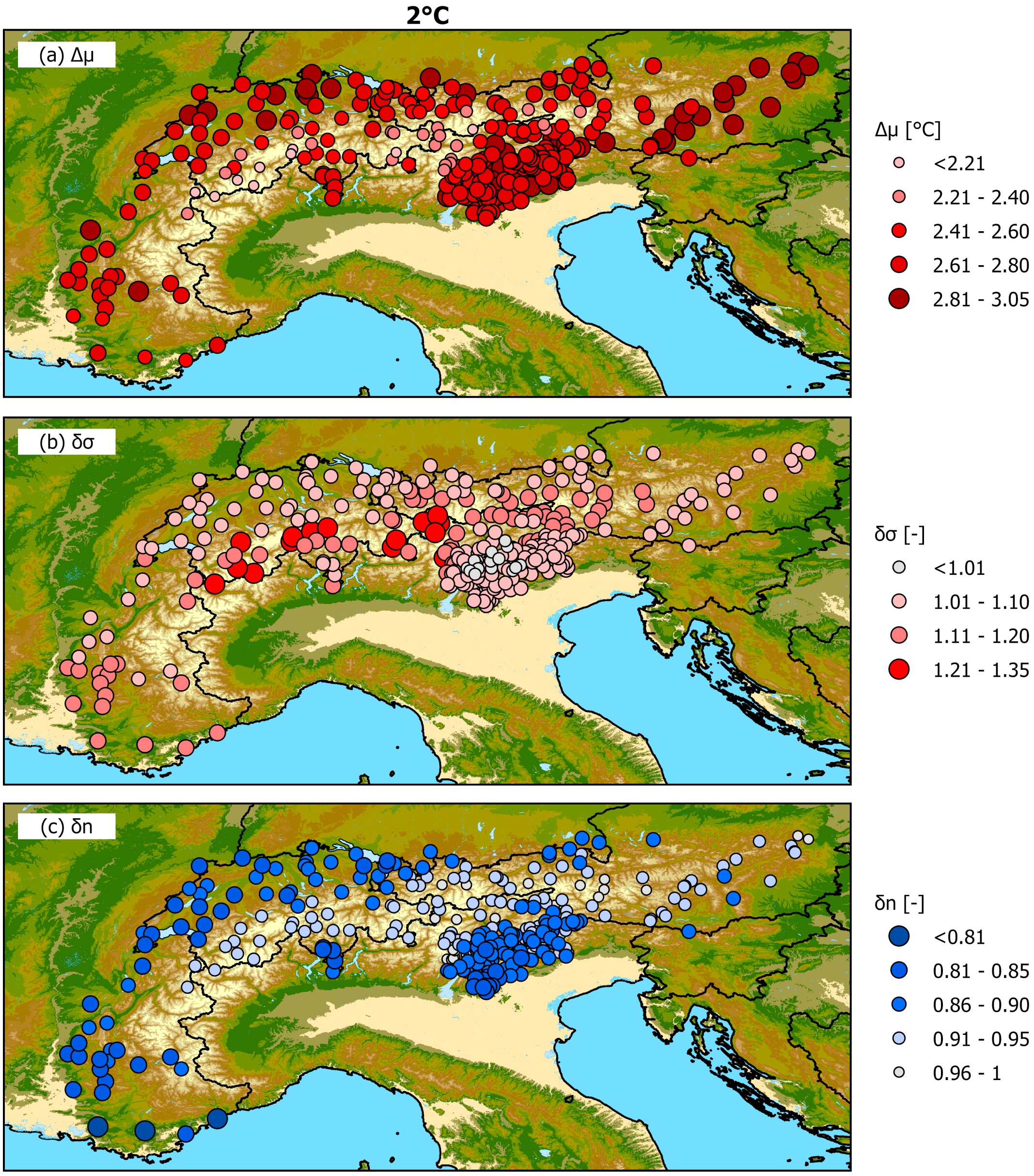

Fig. S4 Median changes in $\Delta\mu$, $\Delta\sigma$, and δn for the 3°C increase in regional temperature.

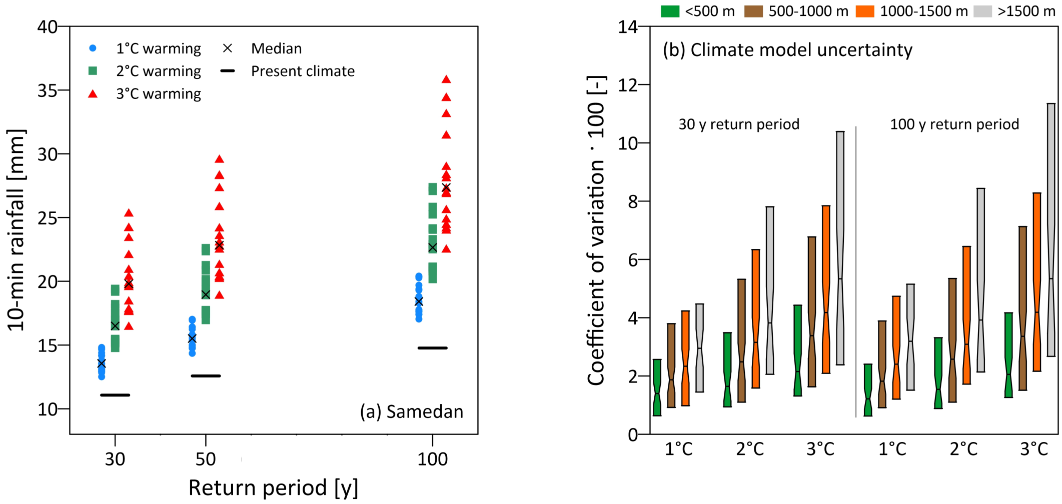

Fig. S5 Climate model uncertainty for (a) individual station (Samedan) and (b) all stations as a function of elevation.
